# “This is my why.” Exploring the concept of meaningful work in early-career veterinarians across Canada

**DOI:** 10.3389/fvets.2025.1595949

**Published:** 2025-06-13

**Authors:** Emily Morabito, Andria Jones, Tipsarp Kittisiam, Martin Cake, Caroline Ritter

**Affiliations:** ^1^Department of Health Management, Atlantic Veterinary College, University of Prince Edward Island, Charlottetown, PE, Canada; ^2^Department of Population Medicine, Ontario Veterinary College, University of Guelph, Guelph, ON, Canada; ^3^School of Veterinary Medicine, Murdoch University, Perth, WA, Australia

**Keywords:** meaningful work, positive psychology, veterinary wellbeing, meaning in veterinary medicine, early career veterinarian, transition period

## Abstract

**Introduction:**

The veterinary profession is marked by significant mental health challenges, including stress and burnout, particularly among early-career veterinarians. Positive psychology highlights the importance of meaningful work as a pathway to enhance well-being (e.g., increasing resilience through a sense of purpose). Despite this, there is limited understanding of how early-career veterinarians perceive and experience meaningful work during the transition from student to practitioner.

**Methods:**

This study aimed to: (1) explore early-career veterinarians’ perceptions of meaningful work, and (2) investigate the intricacies of meaningful work experienced by early-career veterinarians during the transition period. A qualitative, phenomenological approach was utilized, involving semi-structured interviews with 21 early-career veterinarians from Canadian veterinary colleges. Data were analyzed using template analysis to identify themes in the data.

**Results:**

The analysis identified five key themes: the obscure concept of meaningful work, meaning found through connection, making a difference, the ability to be creative and problem-solve, and the evolving nature of meaning. Participants initially found meaningful work difficult to define, reflecting its subjective nature. Connections with animals and humans, such as clients and colleagues, emerged as crucial sources of meaning. Making tangible differences for animals, caretakers, and the community was seen as central to their work. The ability to creatively solve problems provided intellectual satisfaction and enhanced their sense of purpose. Participants noted that their understanding of meaningful work evolved with experience, highlighting the interplay between professional growth and personal fulfillment.

**Discussion:**

In conclusion, early-career veterinarians derived meaningful work from a combination of deep connections, impactful contributions, and creative problem-solving, which evolved over time. These insights can inform strategies to improve well-being within the profession by fostering supportive environments that encourage reflective practices, personal growth, and pathways to meaning and purpose.

## Introduction

Veterinary medicine can be a rewarding profession but is also one marked by mental health challenges (1, 2). Veterinarians experience a higher prevalence of stress, burnout, compassion fatigue, and suicidal ideation when compared to the general population (3, 4). These issues are particularly pronounced among early-career veterinarians who may have not yet adapted to challenging aspects of the profession such as long hours, emotionally taxing client interactions, and the pressures of clinical decision-making (5, 6). Female veterinarians are reported to be more susceptible to these struggles, experiencing higher levels of emotional exhaustion and stress compared to their male counterparts (4, 7). While these challenges exist, many veterinarians also report deriving deep fulfillment from their work, highlighting the importance of understanding what fosters positive well-being in this profession (8).

In recent years, positive psychology has emerged as a valuable framework to help address mental health in veterinary medicine by focusing on enhancing well-being through strengths, positive emotions, and resilience rather than solely mitigating negative mental health outcomes (8). Positive psychology concepts, such as purpose and meaning, are critical for helping early-career veterinarians cope with challenges in their work and develop a stronger professional identity (9). Professional identity formation, blending personal and professional values, has been linked to improved well-being, with meaningful work playing an especially important role (10, 11).

The exploration of meaningful work in veterinary medicine is a worthy pursuit as meaningful work has been associated with greater job satisfaction, resilience, and mental well-being in other fields [e.g., (12)]. For veterinarians in an Australian study, finding purpose and meaning in work has been reported to stem from making a tangible difference in the lives of animals and their caretakers and contributing to broader societal goals such as animal welfare and public health (8). However, defining and experiencing meaningful work can be particularly elusive for early-career veterinarians, who often begin their careers with a deep passion for animals but soon face the realities of practice (13), which may interfere with their realization of purpose and meaning. Because of these complexities, exploration of meaningful work in veterinary medicine is important. Veterinarians who can link their daily tasks to a larger purpose, such as improving animal welfare or educating clients, are more likely to feel personally fulfilled (7) and experience the myriad benefits that living a professional life of meaning affords (14).

While meaningful work is known to positively impact well-being, how veterinarians across Canada perceive meaning in their work—particularly during the challenging transition from student to practitioner—remains unclear. To address this gap in knowledge, the objectives of this study were to: (1) Explore early-career veterinarians’ perceptions of meaningful work, and (2) Investigate the intricacies of meaningful work experienced by early-career veterinarians during the transition period.

## Materials and methods

### Theoretical framework

A qualitative, phenomenological approach (15, 16) was employed to explore how participants experience and make sense of meaningful work in their professional lives. This approach is well-suited for investigating lived experiences, like how veterinarians understand and navigate the concept of meaning in their careers. We adopted a constructivist paradigm, which views knowledge as being co-constructed between researchers and participants through reflective dialogues, making it particularly useful for examining a deeply personal and abstract concept like meaningful work (17).

### Participant recruitment and description

Participants were early-career veterinarians (1–7 years post-graduation) who had graduated from Canadian veterinary colleges, were practicing in Canada at the time of data collection and were fluent in English. This transition period is longer than the frequently cited first five years in practice (18), but we wanted to include some veterinarians that transitioned before and after the COVID-19 pandemic, which had significant impacts on both mental health and the veterinary profession’s dynamics (19, 20). Recruitment efforts used convenience sampling and targeted various channels, including veterinary colleges, the Canadian Veterinary Medical Association (CVMA) newsletter, and other professional networks (e.g., provincial VMAs, VetStrategy, alumni networks). We interviewed 21 veterinarians between November 2022 and September 2023. The study population consisted of a diverse representation across demographic factors. In terms of gender, the majority identified as women (*n* = 13), followed by men (*n* = 2), non-binary individuals (*n* = 2), and 4 participants did not to disclose their gender. Geographically, the participants were spread across several provinces: Ontario (*n* = 7), British Columbia (*n* = 6), Alberta (*n* = 4), Manitoba (*n* = 1), and the Maritime Provinces (*n* = 3). Regarding veterinary practice type, the majority were involved in small/companion animal practices (*n* = 13), followed by those in exotics and mixed practices (*n* = 4), mixed (small and large) practices (*n* = 3), and one participant in equine practice. Participant recruitment concluded once thematic saturation was reached; this was deemed to have occurred when the identified themes related to meaningful work were sufficiently developed and robust (21, 22).

### Data collection and analysis

While some phenomenological approaches recommend small sample sizes to enable detailed idiographic analysis, other variants allow for greater flexibility depending on research aims (23, 24). In this tradition, sample size is guided not by fixed conventions but by the nature of the research question and the need to capture shared patterns of lived experience across a range of perspectives (25, 26). Our sample of 21 early-career veterinarians reflects this intent: to generate thick description and identify recurring themes without losing sight of individual nuance. As Bartholomew et al. (23) argue, while smaller samples can support coherence, sample sufficiency in phenomenological work is best determined pragmatically, in alignment with the study’s aims and analytic depth.

A semi-structured interview guide (Appendix 1) was designed to elicit narratives about participants’ experiences, with a particular focus on meaningful work. The guide was refined iteratively based on participant responses, ensuring that emerging topics were explored in subsequent interviews (27). The interviews were recorded and transcribed verbatim, with all identifying information removed for confidentiality.

Data were analyzed using template analysis, a flexible form of thematic analysis that emphasizes hierarchical coding while allowing iterative refinement throughout the process (28, 29). This approach aligns with our constructivist phenomenological orientation, which prioritizes understanding participants’ lived experiences and how they make meaning of those experiences (16). An initial coding template was developed from a subset of transcripts and revised iteratively as analysis progressed. A codebook was maintained to document theme definitions and supporting quotations, evolving in depth and structure alongside our understanding of the data. Template analysis was particularly suited to our sample size and research aim: to identify shared experiences across a relatively large group of early-career veterinarians while remaining attuned to individual nuance. Data were managed using Quirkos software (Version 2.5.3) to support hierarchical organization. The final template reflected a hierarchical structure of main themes and more granular subthemes, illustrating the varied yet interconnected ways participants understood meaningful work.

### Reflexivity statement

The lead author (E.M.) was a Ph.D. candidate at the time of the study with experience in the veterinary field. Although not a veterinarian, E.M. had extensive experience working within veterinary education and mental health contexts, particularly in the dairy and equine sectors, which influenced her understanding of meaningful work in veterinary medicine. E.M. is a cisgendered White female and acknowledges the privileges this has afforded her in navigating the space of veterinary medicine. Additionally, E.M. has close relationships with many veterinarians and entered the study with preconceived notions of what meaningful work might be for veterinarians. She engaged in reflexive journaling throughout the research process and actively discussed the codebook with all co-authors to ensure that these perspectives were considered. Co-authors C.R., A.J., M.C., and T.K., are all veterinarians with research experience in veterinary mental health and wellbeing. C.R. brings a strong foundation in veterinary epidemiology and expertise in qualitative methods. Her interdisciplinary background and international experience added to the analysis by ensuring attention to both the structural and interpersonal dimensions of veterinary work. A.J. contributed extensive experience in veterinary mental health research and well-being programming. Her applied work in resilience training and positive psychology, including teaching veterinary students, provided important conceptual grounding for interpreting participants’ reflections on meaningful work and occupational wellbeing. T.K. was a graduate student at the time of this study with expertise in emotional intelligence in veterinarians, which informed her sensitivity to how emotional dynamics and relational experiences shaped participants’ narratives. M.C. is a veterinary educator and researcher whose focus on professional competencies and resilience in the veterinary context supported the team’s analysis of how educational preparation intersects with the development of meaningful careers. All authors contributed insights during the codebook discussions, ensuring that the veterinary-specific scope of meaningful work were captured and interpreted amongst co-authors.

### Trustworthiness and rigor

Several steps were taken to help ensure the trustworthiness and rigor of the study based on criteria developed and defined by Lincoln and Guba (30) and Nowell et al. (31). Credibility was enhanced through participant validation: participants were invited to review their transcripts, with two taking this option, and 16 participants requested and received summaries of the study’s findings to ensure the soundness of their input (32, 33). To support transferability, we collected demographic data from participants, to help interpret the study’s findings within the defined population. An audit trail was maintained throughout the analysis process, including documenting key decisions and iterations, which contributed to the study’s dependability. Collaborative analysis discussions among all authors ensured confirmability, as multiple perspectives were considered during the coding and theme development stages.

This study was approved by the Research Ethics Boards of the University of Prince Edward Island (#6010416) and the University of Guelph (#22-11-027).

## Results

**Figure 1:**
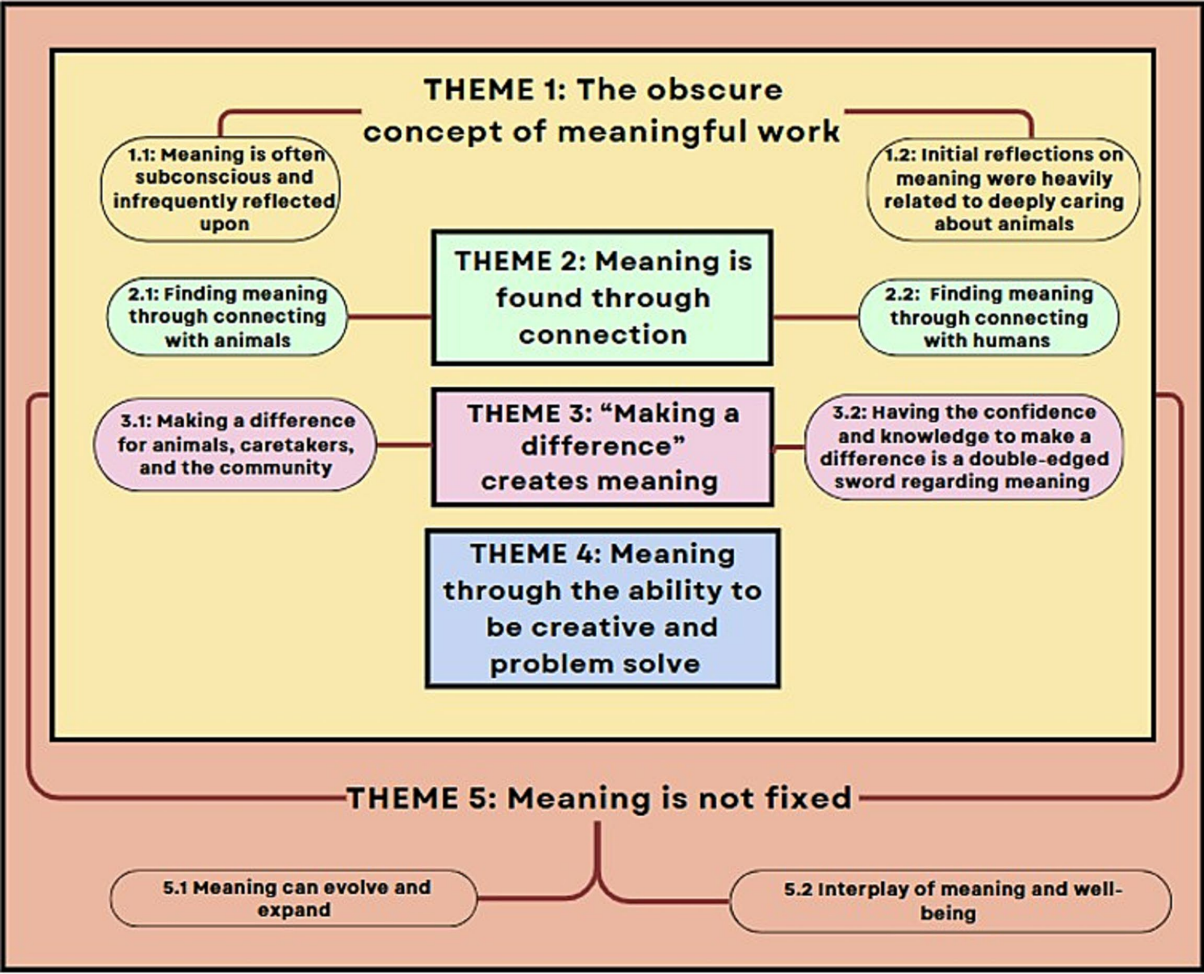
A thematic map visualized from the final codebook, illustrating the hierarchical relationship among the five interconnected themes developed from this study’s analysis. At the highest level, Theme 5, “meaning is not fixed,” encompasses all others, reflecting how participants described meaningful work as fluid, shaped by experience, reflection, and mental well-being. Nested within Theme 5 is Theme 1, “The obscure concept of meaningful work,” which highlights the initial difficulty many participants had in articulating what meaningful work meant to them, underscoring its complexity and subjective nature. Themes 2 through 4—connection, making a difference, and creativity/problem-solving—are situated within both Themes 1 and 5, indicating that these experiences informed participants’ early understanding of meaningful work and continued to evolve over time. The nesting structure conveys how participants described meaningful work as layered, complex, and in constant flux throughout their early careers.

Our research findings yielded five major themes (Figure 1), which are presented below alongside illustrative participant quotes. Participants’ voices are represented using italicized text. To preserve authenticity, quotes were kept as close to the original speech as possible, maintaining the tone, cadence, and informal speech patterns that reflect how participants made meaning of their experiences. To improve readability without altering meaning, we abbreviated some quotes using “[…]” to indicate omitted portions, and square brackets were used to insert clarifying words, where helpful. Minor grammatical adjustments were made in select cases for clarity. To protect confidentiality, all identifying details were removed, and participants were not labeled or numbered, to prevent the association of multiple quotes with specific individuals.

### Theme 1

#### The obscure concept of meaningful work

##### Sub-theme 1.1: Meaning is often subconscious and infrequently reflected upon

During the interviews, many participants seemed taken aback when asked what makes veterinary medicine meaningful to them, or other similar questions related to meaning. The concept of meaning often led to hesitation, with participants responding with long pauses or asking for clarification. For example, some replied, “*Can you elaborate a bit more about that question?*” or *“Um, I do not know, […] nothing’s coming to mind.”*

Despite the interviewer’s reassurance that there were no right or wrong answers in this interview, participants still expressed concern about needing to provide a *“correct*” answer. For example, one veterinarian responded, “*That is a tough question. And I do not know if I have the right answer to it… Or any answer to it.”* Additionally, some participants hesitated to share their thoughts, concerned that their perspective on meaning might differ from others. As one participant remarked, *“That’s a really tough question. Because I think every veterinarian finds fulfillment […] in their work in a different way”*.

Despite several participants’ hesitancy in discussing meaningful work, a few were familiar with the concept of meaningful work and had spent time reflecting on it. These individuals often traced their understanding of meaning back to formative childhood experiences. For instance, one participant shared how their upbringing shaped their sense of meaning:

*“So whenever I was little, my mom would […] tuck us into bed at night. And then she would be like, ‘What did you do today to make the world a better place?’ […] I would be like, ‘Oh, I helped a caterpillar cross like the sidewalk’ or you know […], it was just sort of like, instilled within me from a young age that like, if you can help, […] you should help.”*

##### Sub-theme 1.2: Initial reflections on meaning were heavily related to deeply caring about animals

After the initial struggle to respond to the question about meaningful work, upon reflection, many participants defined this concept by emphasizing their deep admiration for animals. As one participant put it, *“I’ve always loved animals. And I feel like that is the answer everyone’s gonna give here.”* This sentiment was widely shared and seemed to reflect a foundational emotional connection. However, it was not just the love for animals that shaped their sense of meaningful work, but the desire to help them that drove these veterinarians. This desire represented a more purposeful aspect of their motivation, as it aligned with their values and provides direction to their actions. For example: participants often highlighted the *“innocence*” of animals as a key factor that makes their work particularly rewarding. For instance, one veterinarian remarked that *“animals are so incredibly innocent,”* which makes “*serving them*” a “*unique*” and “*fulfilling experience.”* Participants found meaning in the simplicity and innocence of animals, contrasting them with the complexities of human experiences. As one veterinarian observed, *“… they love us so much. And they are so simple. Like, their needs are complex, but they are more simple than people are.”* This appreciation for the innocent and simplistic essence of animals appeared to form a central pillar in how these early-career veterinarians perceived meaningful work.

Additionally, participants’ sense of responsibility was amplified by the understanding that “*an animal does not have someone who will advocate for their best interests, […] that person ends up being me, I find that super meaningful,”* and some participants drew parallels between their work with animals and pediatric care. For example:

*“And I kind of I think a lot about, like pediatrics in that sense, like human doctors. Babies are also so innocent. And I feel like if a pediatrician sees it that way, which I’m sure they do, that they can help this vulnerable population and just like we can help the animals, it’s a really rewarding feeling.”*

For many participants, the core of meaningful work was rooted in improving animal welfare. As one participant explained, *“the meaningful work is to make their welfare as good as possible. That’s like, the whole point, in my opinion.”* The gratification derived from seeing tangible improvements in an animal’s condition further reinforced this perception, as highlighted by another participant: *“Whether or not I can actually fix the problem, I think if I can see, you know, based on my clinical assessment, that they seem to be feeling better […], that to me is gratifying.”*

### Theme 2

#### Meaning is found through connection

##### Sub-theme 2.1: Finding meaning through connecting with animals

As the interviews progressed, participants further explored their concepts of meaning. Participants found deep meaning in the unique, non-verbal communication they share with animals, which allowed them to bypass human-centric communication methods and forge strong bonds with their patients. As one participant noted, *“Animals are instantly easier to connect with because in a way, you take out the verbal aspect, there’s the body language, the energy that’s there, the connection.”* Another veterinarian added, “*You can connect and just feel connected to them. Even if you cannot talk, there are ways to communicate without language*”.

However, the non-verbal connection was also described as a hindrance to diagnosing and treating animals for participants. The absence of verbal communication, while creating the perception of a unique bond for participants and connecting them with meaning, made it difficult to ensure the animals receive the help they need. One participant pointed out, “*They do not have a voice, and they cannot tell people what they are feeling or what they need,*” which participants indicated can make diagnosis more “*difficult*” and “*frustrating.*” Further, handling “*really fearful [animals]*” made effective diagnosis and treatment challenging, and masked participants’ connection with the animals, and thus, their sense of meaning. Although these behaviors were understood as *“not malicious,”* participants still struggled with the inability to communicate their intentions to help the fearful animals. One veterinarian expressed this frustration, saying, “*I cannot explain to them that I’m trying to help […]. If only we could explain that it’s for their good, you know. And so that makes it a little bit more challenging to directly see how I’m helping the animal*”.

To find meaning in working with fearful animals and overcome the challenges of non-verbal communication, participants sought new ways to develop meaningful connections and improve the experience for everyone involved. This often included trying to understand the animal’s perspective or creating a mutual understanding with the animal to establish a safe environment where the animals would not fear them. One participant explained, “*You can read how they [an animal] feels in the environment […] and it’s very rewarding or fulfilling when you can tell one takes a liking to you, or feels safe with you.*” Participants also emphasized that education and training in areas such as “*fear-free*” animal handling, focusing on “*minimal restraint*,” “*longer appointment times,*” and “*practicing extreme patience*” have been crucial in connecting them to their sense of meaning. As one veterinarian shared, “*I think that has made an incredible difference in the experience that the animals have in the clinic.*” These efforts have led to rewarding moments, including instances where “*it’s pretty cool to have a pet recognize you and get excited to see you when you are a scary doctor. It’s pretty neat that we can make their experiences better*”.

##### Sub-theme 2.2: Finding meaning through connecting with humans

While participants described their initial motivation for entering the profession to be a love for animals, the transition from student to practitioner made it clear to participants that working with human counterparts was a significant part of their job. The transition period brought to light that connections with coworkers and “*being a member of a team*” were crucial sources of meaning, particularly because coworkers experienced many of the same challenges. For example:

“*…having those people that you can talk to about these things [work experiences], and who will lift you up …I think that is another really, really important aspect of trying to continue to recognize that what you are doing makes a difference*.”

However, the transition from student to practitioner also exposed the complexities of client interactions. One participant highlighted that “*the client communication, the client interactions, not all of them are positive, a lot of them are actually negative. It is the hardest part about being a vet*.” Participants reported how these negative interactions challenged the sense of meaning in their work, especially when clients failed to understand or support the veterinarian’s efforts. As one participant described, situations where clients were unwilling or unable to follow medical recommendations for their pet thwarted their sense of meaning, “*when we cannot do something it’s… I feel like you’d lose that meaningfulness… like if your words aren’t being heard, if you are just talking to a stone wall, what’s the point?*”

While frustrating client interactions were frequently described, some participants also described finding meaning in building relationships with clients, emphasizing the importance of trust. For example, in discussing what helped with their connection to meaning, a participant shared:

“*I have quite a few longer-term clients, even now that I’m going between clinics, who we have kind of built a relationship and rapport together, […] we trust each other, which I think makes a big difference.*”

Respect and gratitude from clients also contributed to a sense of meaning, with one participant recalling:

“*… […] you get emails from owners, I had one last week, and it just kind of hit me, really, really, kind of made me emotional. And it was […] ‘thank you so much for being able to provide the care that you did for our senior dog. We so appreciate what you have done’ […] And it just, you know, it kind of it gets you in a way that you go okay, well, this—this is my why.*”

Moreover, participants recognized a shared bond with owners—a mutual love for animals—reinforcing their ability to connect with owners. This connection with clients, centered around the welfare of their pets, often provided meaning, as another participant reflected:

“*But I do like people when it comes to the common denominator of their pets. So that is what I can connect with them on. […] Helping people and bringing it back to the patient itself is what gets me through a lot of the days.*”

Ultimately, observing clients interact with their animals allowed for reflections on how caretakers coexist with their animals, providing a sense of connection to clients, and thus, meaningful work:

“*It’s like that animal bond between people, it’s different for everyone, because everyone treats their pets differently. But I think it’s just an extension of people wanting to take care of something that they know cannot take care of themselves. And it’s just an extension of […] human compassion.*”

### Theme 3

#### “Making a difference” creates meaning

##### Sub-theme 3.1: Making a difference for animals, caretakers, and the community

The concept of “making a difference” beyond the individual animal patient was frequently described as a key aspect of meaningful work for early-career veterinarians. One participant captured this sentiment by stating:

“*It’s like when I feel like I’ve made an actual difference both to the animal and the owner in terms of like their short-term quality of life. I think, especially in in the field that I’m working in, helping the humans is just as important. […] I think that you can usually tell when an owner feels like you have […] made a difference. And I think that for me is probably what is most meaningful.*”

Although the connection to, and working with humans, was not initially a part of the descriptions of personal meaning for many participants, when further discussing the impact of their work, many described finding meaning through making a difference for both patients and their caretakers. The idea that making a difference extended beyond the animals became evident when participants noted how much effort and resources many clients invest in their animals. As one participant explained, “*[clients are] spending all this money and [putting] time […] into their animals. And then we are able to actually make a difference there.*” The sense of “*making a difference”* was further reinforced through the gratitude veterinarians received from clients, as discussed in Theme 2. One participant shared:

“*Sometimes they’ll even write me a card or something like that, like, that really gives me a huge boost, and just pushes me forward knowing that, you know, at least in that case, I was able to make a difference [to that client].*”

Clients’ expressions of thanks and recognition of veterinarians’ efforts were described to help participants feel their services were more tangible and meaningful. Further, for some, the feeling of making a difference extended beyond the immediate scope of their clinical practices to include helping families and even broader communities (i.e., volunteering at shelters). As one participant expressed, “*I feel like I’m helping the community, the animals owned or not, and you know, their families*”.

##### Sub-theme 3.2: Having the confidence and knowledge to make a difference is a double-edged sword regarding meaning

Early-career veterinarians described experiencing a lack of confidence at the start of their career in practice. As one participant described, “*So you get thrown into things way too quickly. And then you are kind of … I was kind of left feeling really overwhelmed and really inadequate.*” This sense of inadequacy was exacerbated by a shortage of veterinarians (“*because like we need more vets*”), a reality that placed additional pressure on new practitioners to use the knowledge they obtained to fill the current gaps in veterinary medicine, even though they lacked confidence. As one participant explained, *“this place would be closed otherwise, if I wasn’t here*.” This shortage created a pressing need for new veterinarians to quickly apply their knowledge, intensifying the responsibilities they faced. Some participants were able to find meaning through this sense of responsibility and the fact that they have the power to do this job. For example, this participant described their view on responsibility.

“*And I was reading a book a few months ago that was talking about with, with skill and ability comes responsibility. So I think I see it more as I’ve done the school, I’ve done the training, I have these skills. And thus, I’m responsible to serve the animals and the people in my community.*”

However, other participants described that the burden of high expectations without feeling confident in their skills made their work feel “*less meaningful*” and “*more of a responsibility or a need*.” This perception then led to a disconnect with their sense of “*making a difference*” and *“finding meaning”*.

As participants gained experience, they were able to improve finding meaning in their ability to make a difference through the knowledge they had acquired, making application of this acquired knowledge more of a meaningful experience instead of a burden. One participant expressed this growth in confidence, saying:

“…*feeling like I actually know things like […] when I’m able to answer questions that clients have, um, it makes me feel like I, like I’m important, and I do make an impact on these people’s lives.*”

This growing confidence and competence served as a continual reminder of their purpose, as another participant shared:

“*This is what I’m here for, this is my job. And I’m able to provide these services that nobody else can provide. So it definitely made it an overall better experience and kind of reminded me why I was doing it.*”

Through this early-career evolution, some participants moved from feeling disconnected from meaning as a result of their feelings of overwhelm in the transition period to recognizing the significant impact they could make, finding deeper meaning in their roles as they developed their confidence and knowledge.

### Theme 4

#### The ability to be creative and problem-solve

The unpredictable and varied nature of their work was a significant pathway to meaning for many participants. They highlighted that the veterinary profession offers continuous learning and engagement through the need to “*solve puzzles*.” As one participant expressed, “*the problem solving behind practicing good medicine. That’s what makes it meaningful work for me.”* Participants reported that this “*puzzle-solving*” aspect extended beyond medical diagnosis to include financial challenges, with some veterinarians finding meaning in working with clients’ budgets: *“…being able to […] work with owners, and like their goals, and also their budget, which in this field is like super important, makes, like… it provides a healthy challenge for me.”* For some, this aspect of the job, including offering financial relief when possible, contributed deeply to their sense meaning as one participant noted, “*I’ve been able to discount quite a few times that have been super, super meaningful”*.

The daily variability reported by participants kept the work from becoming monotonous and fostered a sense of accomplishment. One veterinarian highlighted this by stating, *“Like … there’s going to be something new every single day. So it’s rewarding to learn every day as well. No day is the same. And I like that.”* Participants found meaning through the intellectual stimulation that came from facing novel cases and situations that pushed them out of their comfort zones. As one participant acknowledged, “*I do like that it’s difficult. I do like that I see things where I’m like, holy moly, I really do not know what to do, I’ve never seen this before*.” This unpredictability not only kept the work interesting but also encouraged meaningful creativity (e.g., treatment options for different budgets), with another participant stating, “*So I think that kind of creativity was really, it blew my mind how creative I could be.*”

The necessity to adapt and problem-solve in unique scenarios revealed the creative potential within the profession, which participants reported to make their work more fulfilling and meaningful. The ability to navigate clients’ financial challenges while still providing high quality care added another layer of complexity to the problem-solving process, further enhancing the sense of achievement.

### Theme 5

#### Meaning is not fixed

##### Sub-theme 5.1: Meaning can evolve and expand

Some participants described the concept of meaningful work as having the capacity to shift and evolve over time. Participants acknowledged this evolution, with one stating, “*What is meaningful work? I think that this question actually, kind of has changed since I was in school,*” and another noting, “*I think more time in the field will help me, like figure that out as well.*” A shift in meaning often included a growing appreciation for the human side of the profession—which some veterinarians initially overlooked to focus on their connection to animals—but later found rewarding. As one participant reflected, “*I think, along the way, I started to get a lot of value out of helping the people as well*”.

Additionally, participants believed that their work as a veterinarian influenced other aspects of their lives, contributing to a broader sense of meaning beyond their occupation. One participant shared:

“*… I mean, working with so many different people, like every day has definitely improved my communication skills. And that’s also had a positive impact in my everyday life as well like with friends and family and other people. So that’s been a really good meaningful change in my life from being in that environment as a vet*.”

This integration of professional skills into personal life demonstrates how meaning can expand beyond the immediate context of work for participants.

Overall, participants described how ongoing exposure and experience in the field helped them refine where they feel most needed and where they can make the most impact. As one participant put it, “*Yeah, and the more that you are exposed to [additional experiences] […] the more that you start to figure out where, where you feel like you are needed, and where you can make the most difference.*” A key aspect of this evolving sense of meaning, according to participants, was learning to reflect on their work from different angles, transforming their perception of meaning:

“*So it’s, it’s, for me, it’s about trying to like, find the good things in each day that happened … I think part of it is how you frame it, like just mentally…I like to reflect on the stuff that goes really well or like when I absolutely nail a diagnosis and things go awesome. I try to do try to remember those cases*.”

Through these reflections, these participants were able to adapt their understanding of meaningful work and find new sources of fulfillment as they progressed in their careers.

##### Sub-theme 5.2: The interplay of meaning and mental well-being

Within participants’ broader understanding that meaningful work was not fixed, participants outlined how their mental well-being played a complex and influential role in how they experienced and perceived meaningful work. For instance, one participant reflected on this complexity by stating, “*Hmm… that’s tough. Um, I think. I think we need to be well enough to be able to enjoy our work. I think that’s like a building block, a basement building block of finding meaning*,” suggesting that a baseline level of mental well-being is essential for engaging with work meaningfully. However, the relationship between well-being and meaning was often portrayed as complicated. For example, this participant described how their mental state can fluctuate, impacting their perception of meaning:

“*I’m usually pretty positive, and […] I feel like, I certainly am making an impact and like, my role is important. And then there’s been other days, like, maybe after a stressful day […] I certainly do have thoughts where I’m like, ‘What am I doing? Like, why? Why am I like, trying so hard to like, make a positive impact in this animal’s life and, you know, giving these clients all this information to help them make a decision […] when at the end of the day, they are just like, going to be angry at me, no matter what I do, then like, then I certainly get quite like anxious about it, and like, stressed out and I do start to think about, like, Do I even have an impact?*”

This fluctuation that this participant experienced illustrates the impact of stress and negative interactions on their sense of meaning.

Participants also emphasized the significant role their work environment played in both meaning and well-being. One veterinarian pointed out, “*If you feel like you are going to a war zone every day, it’s probably not going to help with making your work feel meaningful*” highlighting how a negative work environment can erode the sense of meaning via jeopardizing well-being. On the other hand, a supportive team and leadership were also mentioned as vital components of meaning, as one veterinarian shared, “*And then having a supportive workplace. And a boss that is willing to do communication seminars, and team building. And all of that was also very positive and kind of kept me going.*”

There were moments when participants found that their sense of meaningful work could outweigh their personal struggles. One participant expressed this resilience, saying, “*I feel like no matter how, like tired I am, and there was at one point, I was feeling like, on the verge of like, compassion burnout [sic], I still felt like my work was incredibly meaningful.*” Even in situations where outcomes were not favorable, participants reported that the work often still retained its meaning (e.g., “*So even though it did not turn out well, that still felt very meaningful*”). Ultimately, while mental well-being was described to influence the perception of meaningful work, as one participant concluded, “*I do not think that my mental well-being changes my definition of meaningful work, but it might change my perception of whether or not I find my work at the moment meaningful*”.

## Discussion

This study explored the understanding of meaningful work among early-career veterinarians, a group susceptible to mental health challenges due to the emotionally and physically demanding nature of veterinary practice (18, 34). Through template analysis, we identified that participants derived meaning from deep connections with animals, making a difference in their communities, engaging in creative problem-solving, and reflecting on their evolving roles in the profession.

Participants in this study initially struggled to articulate what meaningful work entailed, highlighting the concept’s complexity and subjectivity, especially during the transition from student to practitioner. This difficulty aligns with the findings of Both-Nwabuwe et al. (35), who argued that meaningful work requires personalized, situational definitions, particularly for individuals at the start of their careers, as they are still navigating their professional identities and values (35). Moreover, Van der Deijl's (36) exploration of meaningful work as a balance between personal well-being and societal contributions resonates with the experiences of the participants in this study, who were working to reconcile their personal sense of fulfillment with their broader goals, such as promoting animal welfare (36). This is in line with Martela and Pessi’s (37) framework, which emphasizes that meaningful work is deeply intertwined with both self-realization and a broader purpose. In their model, work is meaningful not only when it allows individuals to realize their potential and express authenticity but also when it serves a higher, prosocial purpose. This dual focus is clearly reflected in the experiences of the participants, who navigated their personal growth while striving to contribute to a larger societal good (37). Further, these findings resonate with the work of Huta and Waterman (38), who explored the distinction between eudaimonia and hedonia in the context of well-being. They confirmed that eudaimonia—rooted in personal growth and transcendence—is integral to meaningful work (38). The participants in this study embodied this eudaimonic perspective, as their perceptions of meaningful work were often shaped by a desire for personal growth and a deep sense of purpose, much like the conceptualization outlined by the PERMA model of well-being (8, 39). The integration of eudaimonic and hedonic elements into meaningful work suggests that early-career veterinarians, like the participants in this study, may experience their work as fulfilling both personally and as part of a broader societal contribution (38, 40, 41).

As interviews progressed, participants’ descriptions of meaningful work expanded beyond their fundamental love for animals to include building trust with clients, highlighting the ever-changing and subjective nature of meaningful work shaped by personal, professional, and societal factors. These shifts highlight the importance of understanding the multiplicity of professional experiences, the development of a professional identity formation, and personal and professional self-reflection in connecting with and sustaining meaningful work (10, 11). The emotional fluctuations described by participants, from positive client interactions to challenging moments of self-doubt, accentuate the episodic nature of meaningful work (42). For example, positive feedback and visible improvements in animal welfare bolstered veterinarians’ sense of purpose, while difficult client interactions and professional pressures diminished it. The emotional ups and downs emphasize the need for targeted training in non-technical competencies, such as self-reflection, emotional intelligence and communication skills, to help veterinarians maintain their sense of meaning through challenges (43).

Central to participants’ sense of meaningful work was the desire to “make a difference” for animals, clients, and the broader community, reinforcing Bailey et al.'s (44) findings that meaningful work often stems from personal evaluations of self-worth and societal validation (44). However, challenging client interactions occasionally disrupted this sense of purpose, suggesting that improving client communication skills could help veterinarians sustain their connection to meaningful work in difficult situations. This understanding of meaning evolved as participants developed professionally, echoing Martela and Pessi’s (37) findings that meaningful work integrates self-realization with a broader purpose, and supporting Toubassi et al. (11) and Van der Deijl (36) who emphasize that meaningful work grows as individuals align their personal development and well-being with their contributions to society (11, 36, 37).

Among early-career veterinarians, creative problem-solving was a key contributor to meaningful work, with participants finding intellectual fulfillment in managing complex medical cases and navigating clients’ financial constraints. This adaptability and creativity align with research suggesting that self-realization and continuous learning are essential components of meaningful work (37). Additionally, the development of creativity through introspection, aligning practices with personal values, and building confidence over time further supports this connection (45). As participants gained experience and confidence, their ability to embrace responsibility and make impactful decisions deepened their sense of purpose, highlighting the importance of fostering self-compassion and reflective practices to support professional growth (44). Moreover, engaging in creative problem-solving and overcoming challenges mirrors the creative processes identified in other professional domains, where resilience, adaptability, and sustained engagement with complex problems contribute to long-term professional fulfillment (13, 45, 46). These findings also integrate with the Self-Determination Theory (SDT), which emphasizes the importance of fulfilling basic psychological needs—autonomy, competence, and relatedness—supporting the idea that creativity and adaptability in problem-solving contribute to both professional development and intrinsic motivation (47, 48). In this framework, creativity is closely tied to the need for competence, where the challenge of solving complex problems enhances a sense of mastery and accomplishment, both of which are intrinsic to well-being and professional satisfaction (40). Additionally, Waterman (49) argues that mastery, a key element of eudaimonia, is central to self-realization, reinforcing the connection between problem-solving, personal growth, and long-term professional fulfillment (49).

To help early-career veterinarians connect with and sustain their sense of meaningful work, strategies such as cultivating self-compassion, encouraging reflective practices, fostering supportive work environments that promote creativity, and explicitly encouraging reflection on the meaning of their work can deepen their sense of purpose, resilience, and long-term career satisfaction. First, cultivating self-compassion can aid veterinarians in recognizing that feelings of self-doubt and uncertainty are normal during the transition from student to practitioner, framing these experiences as essential parts of professional growth rather than personal shortcomings. Incorporating self-reflection practices, such as end-of-day reflections on how daily cases align with personal values or professional goals, can deepen their connection to meaningful work. Reflecting on the broader purpose of their work—what makes it meaningful to them—can further strengthen this connection and clarify their motivations. Clinics can further support this by displaying thank-you notes, positive client feedback, and success stories in shared spaces to serve as visible reminders of their positive impacts. Additionally, reframing challenges as opportunities for creative problem-solving during difficult cases could help foster adaptability and reduce feelings of overwhelm. Establishing mentorship programs and peer support networks can also provide early-career veterinarians with guidance, reassurance, and shared strategies for maintaining meaning in their work (46, 50, 51). Together, these strategies can strengthen veterinarians’ sense of purpose, resilience, and long-term career satisfaction.

While many of our findings may seem intuitive to those embedded in veterinary practice, such as the emotional labor of client communication or the disillusionment with idealized career expectations, they also point to deeper structural and cultural tensions. Participants often entered the profession with a strong connection to animals but later described feeling emotionally eroded by systemic stressors that have been highlighted in other research, including high client expectations, economic constraints, and workplace demands (52–54). These included fee disputes, value misalignments with clients or employers, and feelings of moral distress when ideal care was unattainable due to financial limitations (55, 56). These tensions reflect cultural influence of meaning-making in professional life, where personal fulfillment is mediated by external systems that can both enable and erode meaning and purpose (57–59). Although positive psychology interventions have been shown to improve well-being and build resilience in both general and professional populations (60, 61), we strongly caution against a tendency for its reductive application as an individual solution to systemic issues. A “think positively” framing may unintentionally suggest that distress stems from personal inadequacy rather than contextual strain. Future research and education efforts should therefore consider how institutional systems, cultures, and economic structures shape veterinarians’ capacity to sustain meaningful work.

We acknowledge that participants who chose to participate may have been inclined to reflect on veterinary well-being or have had stronger feelings about the topic than those who did not. The study also relied on participants’ self-reported experiences, which may not always precisely capture their thoughts or behaviors at the time of the interview, due to the inherent challenges of retrospective recall. The focus on a specific sample of Canadian veterinarians, primarily working in companion animal practice, further limits the extent to which these findings apply to veterinarians outside of this study, especially in other countries or cultural contexts where the nature of veterinary practice may differ. Future research should aim to include more diverse samples across different countries and practice settings to capture a wider range of experiences, potentially including quantitative means to facilitate larger sample sizes and generalizability. Longitudinal studies in particular could provide deeper insight into how veterinarians’ perceptions of meaningful work change throughout their careers, and as they encounter different professional challenges and milestones. Additionally, investigating how workplace culture and structured interventions—such as reflective practices, self-compassion training, and mentorship programs—impact meaningful work could provide actionable strategies for enhancing well-being and long-term satisfaction within the veterinary profession. In advocating for reflection as a pedagogical strategy, we also acknowledge the contested role of reflection in higher education. While reflective practices can develop self-awareness, they are not inherently transformative, and the concept itself is often vaguely defined and inconsistently applied. As Tight (62) argues, reflection has become a pervasive yet under-theorized solution in education policy and practice (62). We agree that merely defaulting to “more reflection” may be insufficient unless it is explicitly framed, scaffolded, and embedded within a broader structural critique. Future work should evaluate when and how reflection is meaningfully integrated and when it risks functioning as a placeholder for more difficult institutional change.

In conclusion, participating early-career veterinarians in Canada experienced meaningful work through a combination of deep connections with animals, building trust with clients, making a difference in animal welfare and community well-being, and engaging in creative problem-solving. Initially driven by a love for animals, participants’ understanding of meaningful work evolved over time, influenced by their professional experiences. Recognizing and fostering these pathways to meaningful work is essential for promoting meaning, resilience, and well-being within the veterinary profession.

## Data Availability

The datasets presented in this article are not readily available because the research participants did not consent to data sharing. Requests to access the datasets should be directed to Emily Morabito, eamorabito@upei.ca.
