# Supplementary material for: “This is my why.” Exploring the concept of meaningful work in early-career veterinarians across Canada
Source: Front Vet Sci. 2025 Jun 13;12:1595949. doi: 10.3389/fvets.2025.1595949 (PMC12202214; doi:10.3389/fvets.2025.1595949)
Supplement: Supplementary file 1 [file Table_1.docx]

**Semi-structured interview guide**: Supporting positive well-being of early career veterinarians

**Introduction:**

*Introduce yourself*

*Consent statement*

Today we are going to be covering a few topics which mainly focus on positive psychology and your experience as a veterinarian.

Everything you share during this interview will be confidential. You will not be named or have your name associated with what you say.

What we’re conducting is Qualitative Research, which aims to describe the participant’s views and experiences. So, please share your experiences, even if you feel like they are specific and might not be relatable to others. There are no right or wrong (good or bad) answers.

*Before we begin, do you have any questions?*

**Meaningful Work Questions:**

1. Tell us a little bit about yourself and the vet practice you work at?

Probe: Large animal or small animal

2. What about veterinary medicine is “meaningful work”?

Probe: Describe your feelings around veterinary medicine being part of your life’s purpose.

3. Let’s talk about the role of animals in meaningful work, describe your experience working with animals.

Probe: If they jump to the negative- Why do you think you jumped to negative experiences? Could you give an example of a positive one?

Probe: describe how working with animals has made your work more meaningful.

4. Let’s talk about the role of humans (e.g. clients, coworkers, owners) in meaningful work. Describe your experience working with humans (e.g. clients, coworkers, owners).

Probe: If they jump to the negative- Why do you think you jumped to negative experiences? Could you give an example of a positive one?

Probe: describe how working with humans (e.g. clients, coworkers, owners) has made your work more meaningful.

5. We will switch gears a bit to talk about how your mental well-being plays a role in how you feel about your work.

To what extent does your mental well-being impact your view of meaningful work?

Probe: Can you give an example of how your mental well-being has made your work more meaningful?

Probe: Can you give an example of how your mental well-being has made your work less meaningful?

6. In your opinion, how can we help veterinarians find more meaning in their work?

7. What changes can be made in the veterinary profession to better achieve this?

8. What training/discussion could we provide to veterinary students to better help veterinarians find meaning in their work in relation to work with animals? With humans? What would have helped you strengthen these concepts in vet school?

**Emotional Intelligence Questions:**

*Now we’re going to talk about another topic; “emotions”*

9. Please tell me about a time when **you** were experiencing strong emotions at work.

Did you recognize what you were feeling?

Were you able to control your emotions in that situation?

Probe: What did you learn from that experience?

Probe: IF they jump to the negative: Why do you think you decided to tell me about strong negative emotions versus strong positive emotions?

Probe: Please give me an example of a time you experienced strong positive emotions.

10. Please tell me about a time when **your clients/owners** expressed strong emotions while you were at work. Were you able to recognize your client’s emotions?

Probe: How did you react/manage the situation?

Probe: What did you learn from that experience?

11. How do you feel your ability to understand and control your emotions impacts your work as a veterinarian?

Probe: What about your ability to recognize your client’s emotions?

Probe: How does it impact your interactions with clients? Co-workers? Relationships outside of work (*eg* friends and family?)

12. How do you feel your ability to understand and control your emotions impact your mental health and well-being?

Probe: What about your ability to recognize your client’s emotions? Does it impact your mental health?

13. For the previous questions, we have been discussing Emotional Intelligence, here defined as the ability to perceive emotions in oneself, perceive emotions in others, as well as regulate emotions in oneself.

What do you think would help you in terms of improving emotional intelligence?

**Questions related to training/tool:**

14. We discussed several opportunities for the veterinary profession and veterinary medical colleges to help connect veterinarians to their sense of meaning and improve their emotional intelligence. If we were to provide training in these areas, what delivery approaches do you think would work best to help veterinarians learn these skills?

Probe: In an ideal world, what would a training program look like?

i.e., One long day vs several short days

Online vs in-person

Format: App, webinar, etc.

**Cool Down and Summary:**

Provide a summary of the interview.

Does that fairly summarize our discussion today? Did I miss anything or incorrectly capture anything?

Final question - is there anything that you would like to add?

Thank you so much for your time today. Your participation really means a lot to us.
